# Soil Fungal Communities’ Characteristics of the *Lamiophlomis rotata* Root-Zone to Altitude and Their Relationship with Environmental Factors

**DOI:** 10.3390/microorganisms14051083

**Published:** 2026-05-11

**Authors:** Ming Fan, Yaming Yang, Hui Chu, Ping Chu, Qiang Li

**Affiliations:** 1 College of Ecological Environment and Engineering, Qinghai University, Xining 810016, China; yya1963@outlook.com; 2 College of Forestry and Grassland Sciences/Key Lab of Alpine Grassland Ecosystem in the Three-River-Source, Qinghai University, Xining 810016, China; chuhui207@163.com; 3 Qinghai Provincial Key Lab of Adaptive Management on Alpine Grassland, Xining 810016, China; 4 Xining Ecological Environment Monitoring Station, Xining 810008, China; 13997205696@163.com

**Keywords:** *Lamiophlomis rotata*, root zone, RDA, LEfSe analysis

## Abstract

This study aimed to investigate differences and patterns in fungal communities within the root-zone soil of *Lamiophlomis rotata* across varying altitudes. Specifically, it analyzed the characteristics of soil fungal communities at altitudes of 3600, 3800, 4000, and 4200 m and examined their relationships with key bioactive medicinal constituents and soil nutrients. The results indicated that Ascomycota, Mortierellomycota, and Basidiomycota were the dominant fungal phyla in the *L. rotata* root-zone soil, with *Pseudosperma* and *Clavaria* as the predominant genera. The Shannon and Chao1 diversity indices of soil fungi initially decreased and subsequently increased with increasing altitude. At the same altitude, these indices were higher in the root-zone soil than in the non-root-zone soil. Redundancy analysis revealed that available phosphorus was the primary factor influencing fungal communities in the non-root-zone soil. In conclusion, altitude significantly affected the characteristics of fungal communities in root-zone soil, which differed significantly from those in the non-root-zone soils. These findings provide valuable data to support the conservation of *L. rotata* resources on the Qinghai–Tibet Plateau.

## 1. Introduction

Soil fungi are a pivotal component of the soil subsystem and play a crucial role in plant litter decomposition, nutrient transport, material cycling, and enhancement of the soil microenvironment. They are the principal decomposers in soil subsystems [1,2]. Functionally, soil fungi can be categorized into saprophytic and plant-associated groups, both of which participate in numerous soil biogeochemical processes, including carbon and nitrogen cycling and organic matter synthesis and decomposition [3,4]. For example, soil fungi decompose proteins, hemicellulose, cellulose, and lignin [5] and form hyphal bridges between soil particles, thereby indirectly influencing soil aggregate structure [2,6]. Previous studies have indicated that although soil fungi are less abundant than soil bacteria in soil ecosystems, they possess a greater degradation capacity [4]. Therefore, soil fungal diversity and community structure are crucial indicators for assessing soil ecosystem health and stability [2,6] and serve as sensitive markers of changes in the soil microenvironment.

*Lamiophlomis rotata* (*L. rotata*) is a perennial herbaceous plant belonging to the Lamiaceae family endemic to the Qinghai–Tibet Plateau, where it is valued as a medicinal species [7,8,9,10,11]. This species primarily inhabits alpine grasslands and riverbanks at altitudes ranging from 2800 to 5000 m [12,13,14]. Owing to the reduction in suitable habitats caused by global climate change and the overharvesting of wild populations, *L. rotata* has been classified as a List of Grade II Endangered Protected Plants [8,9,10].

Altitude, as a key topographic factor in the mountainous regions of the Qinghai–Tibet Plateau, has significantly reshaped the life forms and ecological niches of regionally distributed plants. The mechanisms underlying plant adaptation to altitude-induced variations in physiological and biochemical processes, as well as metabolism, exert both positive and negative feedback effects on soil subsystems [15]. Research on the root-associated microbial communities of individual plant species is relatively abundant [11], whereas studies focusing on root zone soil are comparatively scarce. However, the soil microbial community in the root-zone was highly susceptible to the influence of plant root exudates. Based on these issues, this study proposes the following scientific hypotheses: (1) Do fungal communities in the root-zone soil of *L. rotata* differ at different altitudes? (2) Do fungal communities differ between root- and non-root-zone soils? Therefore, this study focused on *L. rotata* and analyzed the characteristics of soil fungal communities in its root-zone soil at different altitudes (3600, 3800, 4000, and 4200 m) and their relationships with key bioactive medicinal constituents and soil nutrients. The findings of this study provide data to support the conservation of *L. rotata* resources on the Qinghai–Tibet Plateau.

## 2. Materials and Methods

### 2.1. Study Area

The study area was located in the southern region of Qinghai Province, China. The land use type in this area is grassland (alpine meadow) with sandy, alpine steppe soil [16]. The soil pH ranges from 6.84 to 8.77, whereas the annual mean temperature ranges from −5.6 to −3.8 °C. The annual accumulated temperature ≥ 0 °C is approximately 1130 °C, and the annual precipitation is approximately 445 mm. Precipitation is concentrated between July and September. The annual average evaporation is approximately 1326 mm, and the total annual sunshine duration is approximately 2607 h [16]. The plot information is presented in Table 1.

### 2.2. Plot Setting and Sample Collection

This experiment used a single-factor design, selecting four altitude gradients of 3600, 3800, 4000, and 4200 m, representing four treatments [16]. Three sampling plots exhibiting typical topography and *L. rotata* growth patterns were selected along the same altitudinal gradient, serving as replicates [16]. Diagonal transect lines were established in each sampling area. Ten *L. rotata* plants were sampled along these diagonal transects, and growth was largely uniform across the specimens. The roots of *L. rotata* were excavated with surrounding soil clumps intact. A blade was used to carefully peel back the soil, which was then collected using a brush. The soil root zone was defined as soil within ≤2 cm from the plant root, whereas the soil non-root-zone was defined as soil located >2 cm from the root zone [16]. The collected soil samples were analyzed to determine their basic physicochemical properties and the fungal communities present. Simultaneously, specimens of *L. rotata* were collected to assess its active constituents. The fundamental physicochemical properties of the soil and active medicinal constituents are presented in Table 1 [16].

### 2.3. Determination of Soil Fungal Community Composition and Diversity

Soil Fungal Community Measurement: (1) DNA Extraction and Detection in Soil Samples. (2) PCR Product Acquisition: Primers targeting the ITS5 (5′-GGAAGTAAAAGTCGTAACAAGG-3′) and ITS1R (5′-GCTGCGTTCTTCATCGATGC-3′) regions were used. All PCR mixtures contained 15 µL of Phusion High-Fidelity PCR Master Mix, 0.2 µM primers, and 10 ng of genomic DNA template. Amplification involved an initial denaturation step at 98 °C for 1 min, followed by 30 cycles at 98 °C for 10 s, 50 °C for 30 s, and 72 °C for 30 s, with a final extension step at 72 °C for 5 min. (3) Pooling and Purification of PCR Products. (4) Library Construction and Sequencing. Soil sample sequencing was performed by Novogene Co., Ltd. (Beijing, China), and data analysis was completed on their cloud platform (https://magic.novogene.com/customer, accessed on 2 August 2024–7 August 2024).

### 2.4. Data Processing and Analysis

Data processing and visualization were performed using Microsoft Excel (2016). One-way analysis of variance was conducted using SPSS version 19.0, and redundancy analysis (RDA) was performed using Canoco 5.0 software. All data are presented as mean ± standard error. Analyses of soil fungal diversity, composition, LEfSe, and the FUNGuild database were performed using a cloud platform (https://magic.novogene.com/customer, accessed on 9 April 2026).

## 3. Results

### 3.1. Soil Fungal Diversity Characteristics

Significant differences were observed in the Chao1 index of the root-zone fungi associated with *L. rotata* at different altitudes (Table 2). As altitude increased, the Chao1 index of the root-zone soil fungi tended to decrease. At the same altitude, the Chao1 index for soil fungi in the root zone at 3600 m and 3800 m was significantly higher than that in the non-root zone. No significant difference in the Chao1 index between the root and non-root zones was found at 4000 m and 4200 m; however, the root zone consistently exhibited higher values than the non-root zone. Except for the treatment at 3800 m in the non-root zone (Table 2), no significant differences were observed in the soil fungal Pielou’s evenness and Simpson’s indices. The overall trend in soil fungal Shannon diversity was largely consistent with that of the Chao1 diversity index (Table 2).

### 3.2. Soil Fungal Composition Characteristics

**Figure 1:**
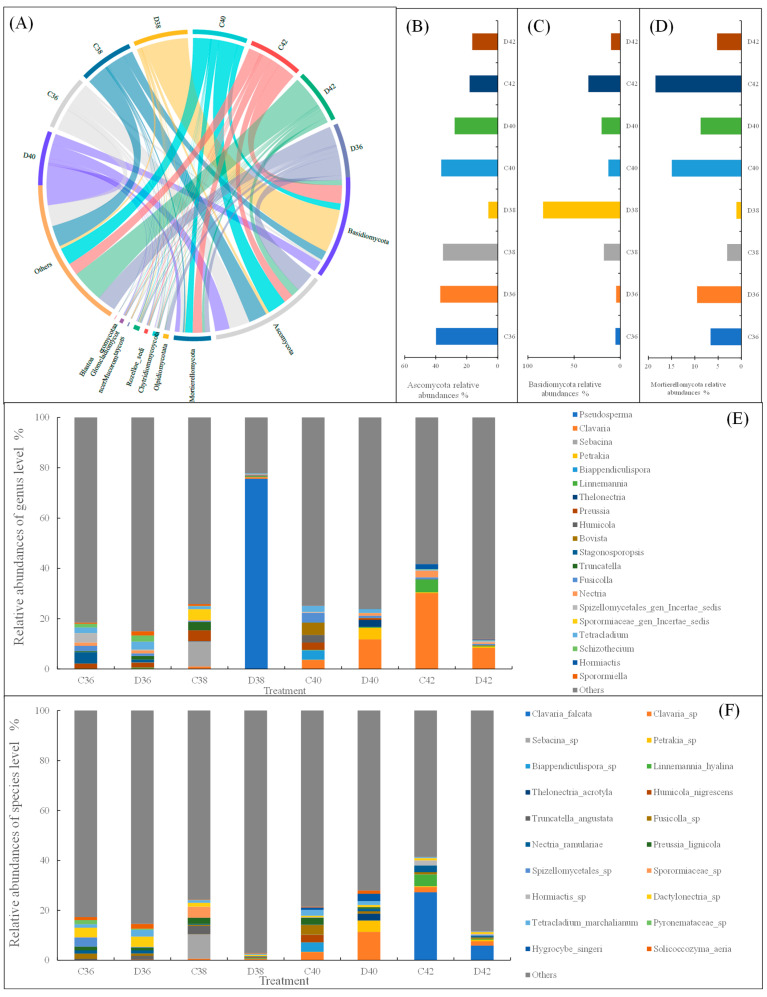
Phylum, genus and species level composition of the soil fungal in *Lamiophlomis rotata* at different altitudes. Note: C: root zone; D: non-root zone; 36: altitude 3600 m; 38: altitude 3800 m; 40: altitude 4000 m; 42: altitude 4200 m. Same below. (**A**) Fungal phyla composition, (**B**) Ascomycota relative abundances, (**C**), Basidiomycota relative abundances, (**D**) Mortierellomycota relative abundances, (**E**) Fungal genus composition, (**F**) Fungal species composition.

The composition of fungal phyla in the root- and non-root-zone soils of *L. rotata* exhibited significant differences across altitudes (Figure 1). As shown in Figure 1A, the dominant fungal phyla were Ascomycota, Basidiomycota, and Mortierellomycota, with average relative abundances of 27.16%, 23.63%, and 8.47%, respectively, collectively accounting for approximately 60% of the total abundance. With increasing altitude, the relative abundance of Ascomycota initially decreased (Figure 1B). At the same altitude, the relative abundance of Ascomycota in the root zone was significantly higher than that in the non-root zone. The relative abundances of Basidiomycota and Mortierellomycota showed no significant variation along the altitudinal gradient or between root and non-root-zone soils (Figure 1C,D).

At the genus level, significant differences in soil fungal genera were observed in the root zone of *L. rotata* at different altitudes (Figure 1E). For example, at altitudes of 3600 and 3800 m, the dominant genera in both root-zone and non-root-zone soils were *Stagonosporopsis* (4.48%), *Tetracladium* (3.20%), *Sebacina* (9.95%), and *Pseudosperma* (75.60%). At 4000 m and 4200 m, the dominant genera in root-zone and non-root-zone soils were *Bovista* (4.88%), *Pseudosperma* (11.81%), *Pseudosperma* (30.15%), and *Pseudosperma* (8.12%). The average genus abundance across treatments indicated that *Pseudosperma* and *Clavaria* were the key genera. At the species level, significant differences in soil fungal species in the root zone of *L. rotata* were also observed among the different altitude treatments (Figure 1F). *Dactylonectria* sp. was the dominant species at 3600 m, whereas *Clavaria falcata* was predominant at 4200 m.

### 3.3. Soil Fungal Differential Species and Ecological Functional Groups

**Figure 2:**
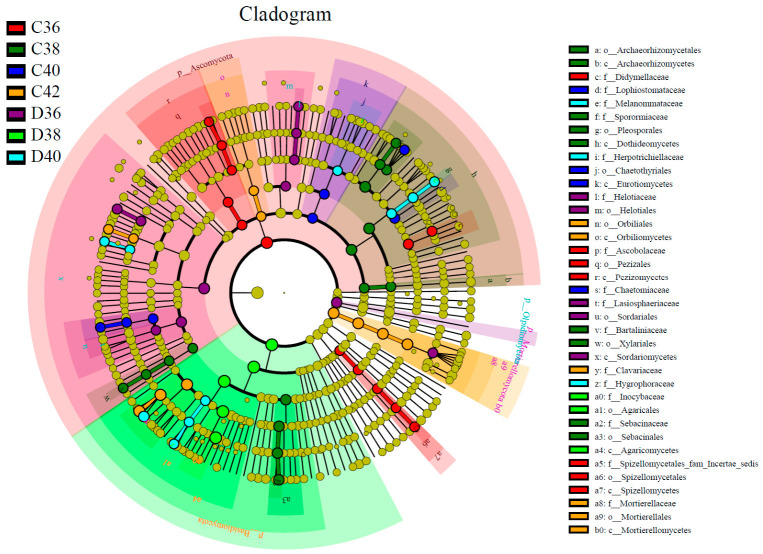
LEfSe analysis of the soil fungal in *Lamiophlomis rotata* at different altitudes.

LEfSe analysis revealed significant differences in fungal taxa within the root zone of *L. rotata at* different altitudes (Figure 2). The number of distinct fungal species in root-zone soils at 3600, 3800, 4000, and 4200 m altitudes was 13, 16, 7, and 10, respectively. In contrast, the non-root-zone soils at these altitudes contained 15, 5, 10, and 0 species. These results indicate that altitude significantly influences the number and composition of soil fungal communities. At the same altitude, except for the 4000 m treatment, the root zone harbored significantly more distinct species than the non-root-zone soil. The identified differential taxa varied significantly in the root zone of *L. rotata at* different altitudes (Figure 2).

**Figure 3:**
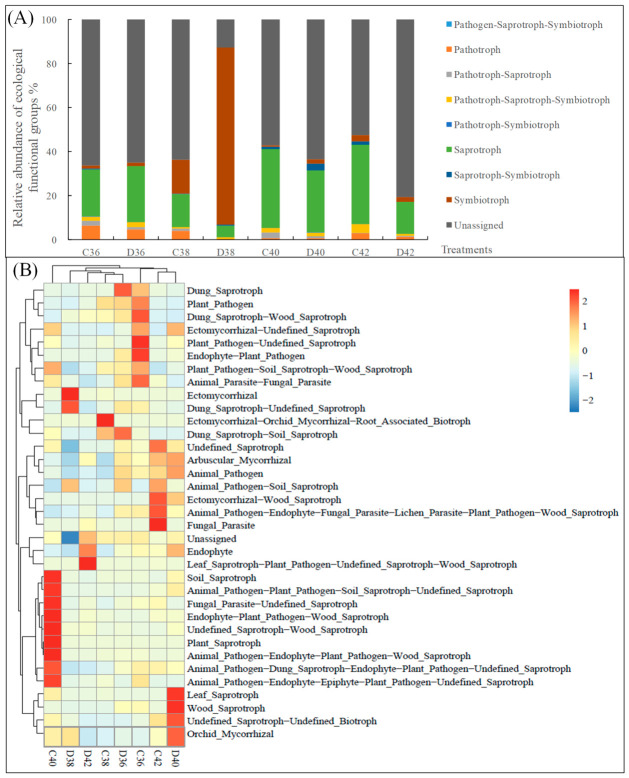
FunGuild feature predictions of the soil fungal in *Lamiophlomis rotata* at different altitudes. (**A**) Relative abundance of ecological functional groups, (**B**) Clustering heatmap of ecological functional groups.

Based on the FunGuild database (Figure 3A), the dominant predictive ecological functional groups in the root and non-root-zone soils of *L. rotata* at different altitudes were saprotrophs (average relative abundance of 22.73%) and pathotrophs (average relative abundance of 13.27%). The relative abundances of saprotrophs and pathotrophs showed significant differences between the treatments but did not exhibit a consistent distribution pattern with increasing altitudes. In addition, cluster analysis of the top 35 ecological functional groups across different *L. rotata* treatments revealed a high degree of similarity in soil fungal ecological functional groups between low and high altitudes (Figure 3B).

### 3.4. Key Soil Nutrient Factors Influencing Fungal Communities

Based on the dominant phyla, genera, and fungal community diversity indices in the root and non-root-zone soils of *L. rotata*, as determined by the results in Section 2.1 and Section 2.2, correlation analyses were conducted with soil environmental factors (Table 3). The Chao1 index of the root-zone soil was significantly and positively correlated with electrical conductivity. The Mortierellomycota phylum in root-zone soil showed a positive correlation with soil moisture content and a highly significant positive correlation with available potassium. In non-root-zone soil, Mortierellomycota exhibited highly significant positive correlations with soil moisture content, available potassium, and available phosphorus (AP). The genus *Pseudosperma* in root-zone soil was significantly positively correlated with pH, soil organic carbon, and 8-O-acetyl-magnolifolide methyl ester. In non-root-zone soil, *Pseudosperma* exhibited a significant positive correlation with 8-O-acetylberberine methyl ester content. The genus *Clavaria* in the root-zone soil exhibited a significant positive correlation with geniposide methyl ester levels.

**Figure 4:**
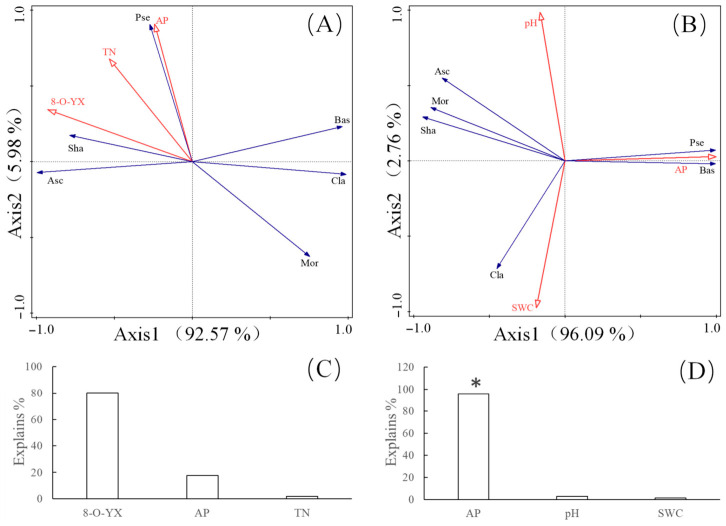
RDA of soil fungal communities and soil factors of *Lamiophlomis rotata.* Note: (**A**) Relationship between root-zone soil fungal communities and soil factors; (**B**) Relationship between non-root-zone soil fungal communities and soil factors; (**C**) Monte Carlo test for root-zone soil fungal communities and soil factors; (**D**) Monte Carlo test for non-root-zone soil fungal communities and soil factors. * *p* < 0.05.

Soil fungal community characteristic indices and soil environmental factors were selected for the RDA. As shown in Figure 4A, the first and second axes of the soil environmental factors accounted for 92.57% and 5.98% of the variation, respectively, with a cumulative contribution of 98.55%. Therefore, soil factors can fully explain the characteristics of root-zone fungal communities. Similarly, Figure 4B shows that the first and second axes accounted for 96.09% and 2.76% of the variation in non-root-zone fungal communities, respectively, with a cumulative contribution of 98.85%, indicating that soil factors also fully explain the characteristics of non-root-zone fungal communities. Monte Carlo permutation tests revealed that 8-O-Acetylgardenin methyl ester had the greatest effect on the fungal community in root-zone soil (Figure 4C), although this effect was not significant (*p* > 0.05). In contrast, AP was a major factor influencing the fungal community in non-root-zone soil (Figure 4D), with a statistically significant difference (*p* < 0.05).

## 4. Discussion

Altitude, as a comprehensive topographical factor, influences climatic variables such as precipitation, temperature, and insolation. These variations suggest that altitude indirectly affects the structure of soil fungal communities [17]. The root zone is a crucial interface between the soil and root microecosystems, impacting plant growth and development via processes such as material transformation and the secretion of trace elements [18]. High-altitude cold regions are typical distribution areas for *L. rotata*. Regional hydrothermal factors play a pivotal role in determining the ecological niche of *L. rotata*. Therefore, understanding the structural characteristics of soil fungal communities in the natural root-zone habitat of *L. rotata* is essential for assessing sensitivity to climate change and vulnerability for the conservation of wild resources.

In recent years, the diversity and distribution patterns of soil microbial communities along altitudinal gradients and their driving factors have garnered significant attention against the backdrop of global climate change [19,20]. Soil fungi, as core members of the soil microbial community, exhibit alpha diversity, which reflects the overall patterns of fungal community evenness, richness, and diversity. Furthermore, alpha diversity can reveal differences in the species composition of microbial communities from different perspectives. Li et al. [17] found that the Chao1 and Shannon indices of fungal communities in alpine meadow soils initially increased and then decreased with increasing altitude, with these indices generally higher in shaded locations than in sunny ones. Hou et al. [21] and Wu et al. [22] reported that soil fungal diversity indices gradually increased with altitude.

Our findings revealed that with increasing altitude, the Shannon and Chao1 indices of soil fungi initially decreased and subsequently increased. At the same altitude, these indices were higher in the root-zone soil than in the non-root-zone soil. However, no significant differences were observed in Pielou’s evenness and Simpson’s indices for soil fungi. Thus, the findings of this study are inconsistent with those of previous studies [21,22]. The discrepancy between our results and those of other studies may be attributed to the specific characteristics of the target species. Since *L. rotata* is a medicinal plant endemic to high-altitude regions, its active constituents and root exudates may influence the diversity of soil fungi in the root zone system under specific conditions. Meng et al. [23] reported that soil fungal diversity indices decreased with increasing altitude, whereas Liu et al. [24] found no significant differences in soil fungal diversity or abundance indices across small-scale altitudinal gradients in alpine grasslands. Considering these variations, differences in the ecological characteristics, topographical factors, and geographical elements of the study site and target species likely contribute to the divergent findings regarding soil fungal community diversity.

In high-altitude mountain ecosystems, altitude over relatively short distances can cause significant changes in environmental factors, such as vegetation and soil, thereby indirectly influencing the composition of soil fungal communities [25,26]. Li et al. [17] found that the dominant fungal phyla in alpine meadow soils at different altitudes were Basidiomycota, Ascomycota, and Mortierellomycota, whereas the dominant genera were *Sebacina*, *Archaeorhizomyces*, and *Preussia*. Zhong et al. [27] reported that the dominant fungal phylum in the rhizosphere soil of flue-cured tobacco at various altitudes was Ascomycota, with relative abundances of 78.87%, 84.14%, 45.20%, and 81.25% at 169.9 m, 383.5 m, 750.8 m, and 1250.4 m, respectively. Our results indicate that Ascomycota, Mortierellomycota, and Basidiomycota were the dominant fungal phyla in the root-zone soil of *L. rotata*, with *Pseudosperma* and *Clavaria* being the predominant genera. Thus, the findings of this study differ from those of prior studies. This discrepancy may be attributed to the unique characteristics of the study site and the target species. As *L. rotata* is a medicinal plant endemic to high-altitude regions, its active constituents and root exudates likely alter the composition of the soil fungal community in its root zone under specific conditions. However, based on the FunGuild database, the dominant ecological functional groups in the root-zone soil of *L. rotata* at different altitudes were saprotrophs and pathotrophs.

Numerous environmental factors influence plant rhizosphere or root-zone microbial communities in varying topography. These factors include soil ecological stoichiometric ratios, soil carbon-nitrogen-phosphorus nutrient content, pH, soil moisture content, bulk density, and topographical features [28,29]. For instance, Li et al. [17] found that organic carbon, total nitrogen, and soil moisture content were the key environmental factors influencing fungal communities in alpine meadow soils in the eastern Qilian Mountains, China. Wu et al. [22] reported that soil pH, nitrate nitrogen content, and total phosphorus content were the primary drivers affecting the distribution of fungal communities in forest soils in Qinghai, China. This study indicates that 8-O-acetylgardenin methyl ester had the greatest effect on the fungal community in root-zone soil, whereas AP was the key factor influencing fungal communities in non-root-zone soil. The primary factors affecting the non-root-zone soil of *L. rotata* at different altitudes were similar to those reported by Wu et al. [22]. The discrepancy between the non-root-zone soil findings and previous studies may be attributed to the effects of the medicinal compound 8-O-acetylgardenoside methyl ester and root exudates.

The findings of this study provide a theoretical foundation for predicting the responses, adaptations, and feedback mechanisms of microbial communities to environmental changes.

## 5. Conclusions

The results indicate that Ascomycota, Mortierellomycota, and Basidiomycota were the dominant fungal phyla in the root-zone soil of *L. rotata*, with Pseudosperma and Clavaria as the predominant genera. The Shannon index and Chao1 richness estimator of soil fungi initially decreased and then increased with altitude. At the same altitude, the Shannon and Chao1 indices of soil fungi were higher in the root-zone soil than in the non-root-zone soil. AP was identified as the key factor influencing fungal communities in non-root-zone soil, while cannot find the key factor influencing fungal communities in root-zone soil. In conclusion, altitude significantly affected the fungal community characteristics in root-zone soil, and fungal communities in root-zone soil differed significantly from those in non-root-zone soils.

## Figures and Tables

**Table 1:** Basic information: Soil physico-chemical indexes and active ingredient content of *L. rotata* [16].

Altitudes (m)	3600 m	3800 m	4000 m	4200 m
latitude and longitude (°)	E101.53840°, N34.86817°	E100.22401°, N34.54389°	E100.42649°, N34.39235°	E100.58044°, N34.33845°
Grassland types	Alpine meadow	Alpine meadow	Alpine meadow	Alpine meadow
ST (°C)	25.00 ± 0.35	23.54 ± 0.51	21.88 ± 0.43	20.48 ± 0.34
SWC (%)	29.12 ± 5.15	31.47 ± 3.84	34.16 ± 3.48	35.83 ± 3.28
EC (µS cm^−1^)	0.06 ± 0.02	0.07 ± 0.02	0.06 ± 0.01	0.03 ± 0.01
pH	8.00 ± 0.05	7.33 ± 0.06	7.28 ± 0.04	6.95 ± 0.02
SOC (g kg^−1^)	34.91 ± 1.85	95.35 ± 4.38	52.13 ± 3.22	21.58 ± 2.63
TN (g kg^−1^)	2.87 ± 0.13	6.58 ± 0.87	3.98 ± 0.39	1.86 ± 0.11
TP (g kg^−1^)	0.66 ± 0.01	1.52 ± 0.05	0.59 ± 0.02	0.70 ± 0.02
AN (mg kg^−1^)	193.47 ± 8.78	482.36 ± 18.72	414.61 ± 6.51	288.8 ± 10.91
AP (mg kg^−1^)	3.81 ± 0.28	15.01 ± 0.86	4.70 ± 0.74	4.26 ± 0.33
AK (mg kg^−1^)	228.58 ± 3.76	162.50 ± 3.22	132.00 ± 3.50	228.50 ± 5.02
SZG (mg g^−1^)	29.18 ± 2.95	22.94 ± 2.07	21.04 ± 1.58	15.54 ± 1.91
8-O-Y (mg g^−1^)	8.61 ± 0.54	9.04 ± 0.58	8.36 ± 0.72	7.00 ± 0.53

Note: ST: soil temperature, SWC: soil water content, EC: electrical conductivity, SOC: soil organic carbon, TN: total nitrogen, TP: total phosphorus, AN: ammoniacal nitrogen, AP: available phosphorus, AK: available potassium, SZG: methyl berberine, 8-O-Y: 8-O-acetylberberine methyl ester. Same below.

**Table 2:** The diversity index of the soil fungal in *Lamiophlomis rotata* at different altitudes.

Altitudes	Root Zone	Chao1	Pielou_e	Shannon	Simpson
3600 m	C	1011.66 ± 111.38 Aa	0.68 ± 0.06 Aa	6.83 ± 0.67 Aa	0.97 ± 0.02 Aa
	D	980.29 ± 31.62 Ba	0.68 ± 0.05 Aa	6.44 ± 0.56 Aa	0.95 ± 0.03 Aa
3800 m	C	834.39 ± 135.23 Ab	0.65 ± 0.08 Aa	6.36 ± 0.87 Aa	0.95 ± 0.05 Aa
	D	541.63 ± 95.94 Bc	0.33 ± 0.11 Bb	3.54 ± 0.17 Bb	0.70 ± 0.08 Bb
4000 m	C	768.38 ± 142.33 Ab	0.62 ± 0.06 Aa	6.14 ± 0.71 Aa	0.94 ± 0.03 Aa
	D	740.96 ± 87.67 Ab	0.64 ± 0.06 Aa	5.92 ± 0.70 Aa	0.93 ± 0.03 Aa
4200 m	C	749.52 ± 101.19 Ab	0.60 ± 0.03 Aa	5.72 ± 0.54 Aa	0.93 ± 0.02 Aa
	D	695.37 ± 132.25 Abc	0.55 ± 0.14 Aa	5.19 ± 0.36 Aa	0.83 ± 0.17 Aab

Note: Uppercase letters denote differences at the 0.05 significance level for root zone at the same altitudes (*p* < 0.05); lowercase letters denote differences in root zone or non-root zone at different elevations (*p* < 0.05). C: root zone; D: non-root zone. Same below.

**Table 3:** Correlation analysis between soil fungal community indicators and soil environmental factors.

Zone	Index	ST	SWC	SE	pH	SOC	TN	TP	AN	AP	AK	SZG	8-O-Y
Root zone	Cha	−0.552	−0.157	0.968 *	0.943	0.946	0.917	0.794	−0.627	−0.883	−0.102	−0.718	0.932
	Sha	−0.68	0.845	−0.017	0.304	0.246	0.146	0.499	−0.728	−0.291	0.924	−0.642	0.363
	Asc	−0.163	0.675	−0.578	−0.283	−0.346	−0.446	0.095	−0.206	0.125	0.745	−0.24	−0.218
	Mor	−0.717	0.987 *	−0.093	0.19	0.154	0.116	0.186	−0.685	0.022	0.999 **	−0.354	0.232
	Bas	−0.038	−0.261	0.428	0.17	0.247	0.401	−0.407	0.102	0.247	−0.399	0.48	0.098
	Pse	−0.667	0.013	0.932	0.975 *	0.965 *	0.914	0.88	−0.753	−0.924	0.085	−0.834	0.977 *
	Cla	−0.78	0.498	0.515	0.758	0.707	0.594	0.905	−0.886	−0.795	0.611	−0.964 *	0.804
Non-root zone	Cha	0.41	−0.099	0.619	0.4	0.436	0.356	0.671	0.487	−0.184	−0.091	−0.644	0.932
	Sha	−0.521	0.904	−0.725	−0.751	−0.745	−0.731	−0.565	−0.637	0.94	0.905	−0.366	0.363
	Asc	−0.479	0.715	−0.876	−0.722	−0.741	−0.667	−0.72	−0.654	0.825	0.712	0.189	−0.218
	Mor	−0.816	0.998 **	−0.861	−0.943	−0.935	−0.939	−0.798	−0.879	0.994 **	0.998 **	−0.475	0.232
	Bas	−0.061	−0.351	0.48	0.257	0.278	0.188	0.238	0.149	−0.5	−0.349	−0.395	0.098
	Pse	0.304	0.083	0.456	0.242	0.279	0.205	0.543	0.352	0.01	0.091	−0.689	0.977 *
	Cla	−0.065	0.591	−0.173	−0.283	−0.26	−0.284	0.012	−0.133	0.59	0.596	−0.564	0.804

Note: ** Indicates a significance level of 0.01, * Indicates a significance level of 0.05.

## Data Availability

The original data presented in the study are openly available in NCBI SRA at number PRJNA1450862 (http://www.ncbi.nlm.nih.gov/bioproject/1450862, accessed on 9 April 2024).
